# Whole-Genome Sequence of *Acinetobacter pittii* HUMV-6483 Isolated from Human Urine

**DOI:** 10.1128/genomeA.00658-17

**Published:** 2017-07-20

**Authors:** Itziar Chapartegui-González, María Lázaro-Díez, Santiago Redondo-Salvo, Laura Alted-Pérez, Javier Gonzalo Ocejo-Vinyals, Jesús Navas, José Ramos-Vivas

**Affiliations:** a Instituto de Investigación Valdecilla IDIVAL, Santander, Spain; b Servicio de Microbiología, Hospital Universitario Marqués de Valdecilla, Santander, Spain; c Red Española de Investigación en Patología Infecciosa (REIPI), Instituto de Salud Carlos III, Madrid, Spain; d Instituto de Biomedicina y Biotecnología de Cantabria (IBBTEC), Universidad de Cantabria, Santander, Spain; e Departamento de Protección Ambiental, Estación Experimental del Zaidín (CSIC) Granada, Spain; f Servicio de Inmunología Hospital Universitario Marqués de Valdecilla, Santander, Spain; g Departamento de Biología Molecular, Universidad de Cantabria, Santander, Spain

## Abstract

*Acinetobacter pittii* strain HUMV-6483 was obtained from urine from an adult patient. We report here its complete genome assembly using PacBio single-molecule real-time sequencing, which resulted in a chromosome with 4.07 Mb and a circular contig of 112 kb. About 3,953 protein-coding genes are predicted from this assembly.

## GENOME ANNOUNCEMENT

*Acinetobacter* species are inherently resistant to several antibiotics or are capable of readily acquiring resistance. Also, clinical isolates are able to rapidly spread among patients and survive in the hospital environment (1). *Acinetobacter baumannii* has been extensively studied because it has been associated with a high mortality rate. However, *Acinetobacter pittii* is increasingly identified as a causative agent of nosocomial infections (2, 3). Moreover, the emergence of carbapenem-resistant *A. pittii* strains possessing carbapenem-hydrolyzing β-lactamases, such as NDM-1, has become a great medical concern (4). The strain used in this study (HUMV-6483) was isolated from urine from a man at the Hospital Universitario Marqués de Valdecilla (HUMV) in Santander, Spain. The strain was routinely cultured in Luria-Bertani (LB) agar or broth at 37°C and frozen at −80°C with 20% glycerol.

A total genomic sample of *A. pittii* strain HUMV-6483 was extracted and purified using the GeneJET genomic DNA isolation kit (Thermo Scientific). The genomic DNA was submitted to Macrogen (South Korea) for PacBio single-molecule real-time (SMRT) sequencing. A single library was prepared for *A. pittii* HUMV-6483 and run on one SMRT cell. With a genome size of approximately 4.07 Mb, PacBio SMRT sequencing provided approximately 100% coverage of the entire *A. pittii* HUMV-6483 genome. SMRT sequencing initially resulted in 223,406 raw reads, with a mean subread length of 6,275 bp (*N*_50_, 7,971 bp), totaling 1,401,914,243 nucleotides. The generated reads were then introduced into the Hierarchical Genome Assembly Process version 3 (HGAP3), which includes assembly with the Celera Assembler and assembly polishing with Quiver (5). The final complete genome resulted in a circular chromosome of 4,070,270 bp with a total G+C content of 39% and a circular contig of 112,604 bp with a total G+C content of 38.5%. A total of 3,953 protein-coding sequences were predicted, of which 74 encode tRNA and 18 encode rRNA. The RAST server (6) predicted coding sequences belonging to 456 subsystems, including 321 involved in carbohydrate catabolism, 303 involved in protein metabolism, 427 involved in the synthesis of amino acids and derivatives, 124 involved in cell wall and capsule synthesis, 133 involved in RNA metabolism, and 91 involved in DNA metabolism, including 260 in cofactors, vitamins, prosthetic groups, or pigments, 99 in nucleoside and nucleotide synthesis, 158 in fatty acid and lipid synthesis, 101 in virulence, 113 in membrane transport, 37 in phosphorus metabolism, 82 in regulation and cell signaling, 5 in secondary metabolism, 37 in phages, prophages, transposable elements, and plasmids, 127 in stress response, and 2 in dormancy and sporulation.

### Accession number(s).

The complete genome sequence of *A. pittii* strain HUMV-6483 has been deposited at DDBJ/EMBL/GenBank under the accession numbers CP021428 (chromosome) and CP021429 (plasmid).
